# Microvesicles Correlated with Components of Metabolic Syndrome in Men with Type 2 Diabetes Mellitus and Lowered Testosterone Levels But Were Unaltered by Testosterone Therapy

**DOI:** 10.1155/2017/4257875

**Published:** 2017-01-12

**Authors:** Jaco Botha, Line Velling Magnussen, Morten Hjuler Nielsen, Tine Bo Nielsen, Kurt Højlund, Marianne Skovsager Andersen, Aase Handberg

**Affiliations:** ^1^Department of Clinical Biochemistry, Aalborg University Hospital, Aalborg, Denmark; ^2^Department of Endocrinology, Odense University Hospital, Odense, Denmark; ^3^Section of Molecular Diabetes & Metabolism, Institute of Molecular Medicine, University of Southern Denmark, Odense, Denmark; ^4^Department of Clinical Medicine, Faculty of Medicine, Aalborg University, Aalborg, Denmark

## Abstract

*Aims.* To investigate how circulating microvesicle phenotypes correlate with insulin sensitivity, body composition, plasma lipids, and hepatic fat accumulation. We hypothesized that changes elicited by testosterone replacement therapy are reflected in levels of microvesicles.* Methods.* Thirty-nine type 2 diabetic males with lowered testosterone levels were assigned to either testosterone replacement therapy or placebo and evaluated at baseline and after 24 weeks. Microvesicles were analysed by flow cytometry and defined as lactadherin-binding particles within the 0.1–1.0 *μ*m gate. Microvesicles of platelet, monocyte, and endothelial cell origin were identified by cell-specific markers and their expression of CD36 was investigated.* Results.* Triglycerides correlated positively with all investigated microvesicle phenotypes in this study (*p* < 0.05), and indicators of hepatic fat accumulation, alanine aminotransferase, and gamma glutamyltransferase correlated with platelet and endothelial microvesicles and CD36-expressing microvesicles from platelets and monocytes (*p* < 0.05). BMI, waist circumference, and fat percentage correlated with CD36-expressing monocyte microvesicles (*p* < 0.05), while insulin sensitivity did not correlate with any microvesicle phenotypes. Microvesicle levels were unaffected by testosterone therapy.* Conclusions.* Metabolic syndrome components and hepatic fat accumulation correlated with microvesicle phenotypes, supporting the involvement of especially CD36 on monocytes in metabolic syndrome pathogenesis. Although testosterone therapy improved body composition measures, microvesicle phenotype levels were unaffected. This trial was registered at ClinicalTrials.gov (NCT01560546).

## 1. Introduction

The metabolic syndrome (MetSy) is constituted by a range of metabolic abnormalities including abdominal adiposity, insulin resistance, hypertension, and dyslipidaemia [1], all of which are associated with an increased risk for type 2 diabetes mellitus (T2D) [2] and cardiovascular disease (CVD) [3]. Given its increasing prevalence and the morbidity and mortality associated with MetSy, there is an urgent need to understand the underlying mechanisms of MetSy.

It is commonly agreed upon that insulin resistance is the central feature in describing the pathophysiology of MetSy [1]. The development of insulin resistance seems highly attributable to an overabundance of circulating fatty acids and ectopic lipid storage [4] leading to inflammation and oxidative stress [5]. Collectively, these alterations contribute to the development of the structural anomalies and cardiovascular complications associated with MetSy, including endothelial dysfunction, atherosclerosis, and thrombosis. Furthermore, low testosterone and sex hormone-binding globulin in men has been linked with the MetSy and T2D [6]. Testosterone replacement therapy (TRT) has previously been demonstrated to improve lean body mass, total fat mass, total cholesterol, and LDL cholesterol in men with T2D [7]. However, a more recent trial could not demonstrate any improvements in the plasma lipid profile nor glycaemic control in T2D patients receiving TRT [8]. Thus, the cardiometabolic benefits with special emphasis on ectopic lipid deposition of TRT remain uncertain.

Changes in levels of certain subgroups of circulating microvesicles (MVs) have previously been ascribed to MetSy [9] and several of its components including insulin resistance, hypertension, hyperlipidaemia, oxidative stress, and waist circumference [10]. MVs are a heterogeneous group of membrane-encapsulated particles that contain cellular components and are released by cells in latent, activated, and apoptotic states [11]. Therefore, a rapidly growing number of studies have recently started to recognize the role of MVs and their potential as biomarkers in a number of diseases. In addition, CD36 is a scavenger receptor and a fatty acid transporter present in most cell phenotypes including platelets [12], endothelial cells [13], and monocytes [14]. Increased levels of circulating CD36 have previously been associated with unhealthy fat distribution [15], atherosclerosis [16], insulin resistance [17], and fatty liver [18], all attributable to ectopic deposition of fat. Thus, measuring the expression of CD36 on MVs could yield important information regarding improvements in the abovementioned components.

The aim of this study was to address the hypotheses that levels of specific phenotypes of MVs correlate with components of MetSy and markers for hepatic fat accumulation and that improvements elicited by TRT affect MV phenotypes in aging, testosterone deficient males with T2D. Testosterone deficient adult males diagnosed with T2D were recruited and randomly assigned to TRT or placebo groups. Circulating MVs of platelet, endothelial, and monocyte origin were quantified in peripheral blood, and their expression of surface CD36 was determined as a surrogate marker for ectopic fat accumulation. Potential relationships were investigated between levels of MV subpopulations and body composition, hepatic fat accumulation, plasma lipids, and insulin resistance prior to the treatment regime, and changes in the levels of MV phenotypes over the trial period were finally compared between TRT and placebo groups.

## 2. Methods

### 2.1. Ethical Aspects

The present study was registered at ClinicalTrials.gov (NCT01560546) and approved by the local ethics committee and the Danish Health and Medicines Authority. All patients gave written informed consent prior to inclusion.

### 2.2. Subjects

This study was conducted on thirty-nine Caucasian men between 50 and 70 years of age, diagnosed with type 2 diabetes within the past ten years, treated with Metformin for ≥3 months, and with bioavailable testosterone levels <7.3 nmol/L. Exclusion criteria were glycated haemoglobin (HbA_1C_) ≥ 9.0%, BMI ≥ 40 kg/m^2^, severe hypertension, cardiovascular, lung, or kidney disease, primary or secondary hypogonadism, known malignant disease, haematocrit > 50%, PSA > 3 *μ*g/L, abnormalities in routine blood tests, nocturia > 3 times/night, significant ECG abnormalities, severe active mental disease, current or previous drug abuse, or the desire to have children [8].

### 2.3. Study Design

This trial was double-blinded, randomized, and placebo-controlled by design and has been described previously [8]. Briefly, subjects were randomly assigned to either TRT or placebo groups and subjected to a 24-week treatment regime with either Testim® 1% testosterone gel or placebo, respectively. Testim 1% testosterone gel, a transdermal testosterone preparation, was supplied in tubes containing 50 mg testosterone each and applied to the shoulders and upper arms at the same time of day throughout the trial period. The initial dosage was 50 mg/day and was adjusted according to plasma testosterone concentration after three weeks to a maximum of 100 mg/day. Subjects were evaluated at baseline and after 24 weeks. During each visit, height, weight, waist and hip circumference, blood pressure, and biochemical characteristics were recorded, and venous blood samples were collected in the fasting state for flow cytometric analysis of MVs. In addition regional body composition was determined by dual energy X-ray absorptiometry (DXA). Insulin sensitivity was determined with the euglycaemic, hyperinsulinaemic clamp technique.

### 2.4. Fluorescent Labelling of MVs

Blood samples for flow cytometric analysis of MVs were collected into tubes containing sodium citrate anticoagulant at a 3.2% (0.105 M) final concentration and the first centrifugation cycle was initiated within one hour after collection. Samples were subjected to a two-step centrifugation protocol to obtain platelet-free plasma (PFP): an initial centrifugation step at 1800 ×g for 10 minutes at room temperature, followed by an additional centrifugation step of the supernatant at 3000 ×g for 15 minutes. PFP was stored at −80°C until analysis. 50 *μ*L of freshly thawed plasma was transferred to TruCount® tubes (BD Bioscience, NJ, USA) in order to allow quantitation of measured events. Samples were incubated for 30 minutes at 4°C in the dark with 5 *μ*L fluorescein isothiocyanate- (FITC-) conjugated bovine lactadherin (83 *μ*g/mL, Hematologic Technologies Inc., VT, USA), 3 *μ*L allophycocyanin- (APC-) conjugated anti-human CD41 (6 *μ*g/mL IgG1, *κ* (clone HIP8, BioLegend, San Diego, CA, USA)), 10 *μ*L phycoerythrin- (PE-) conjugated anti-human CD14 (60 *μ*g/mL IgG2a (clone TÜK4, DAKO, Denmark)), 20 *μ*L PE-conjugated anti-human CD62E (100 *μ*g/mL IgG1, *κ* (clone 68-5H11, BD Pharmingen, New Jersey, USA)), and either 4 *μ*L phycoerythrin- (PE-) conjugated anti-human CD36 (25 *μ*g/mL IgG2a, *κ* (clone 5–271, BioLegend, San Diego, CA, USA)) or 20 *μ*L APC-conjugated anti-human CD36 (6,25 *μ*g/mL IgM, *κ* (clone CB38, BD Pharmingen, New Jersey, USA)), used when appropriate. Isotype controls matching each antibody were used as negative controls. After incubation, samples were diluted in 200 *μ*L Dulbecco's phosphate buffered saline 0.0095 M PO4 (PBS) buffer (Lonza, Basel, Switzerland) that had been filtered through a sterile 0.2 *μ*m Q-Max syringe filter (Frisenette, Knebel, Denmark).

### 2.5. Flow Cytometric Measurement of MVs

**Figure 1:**
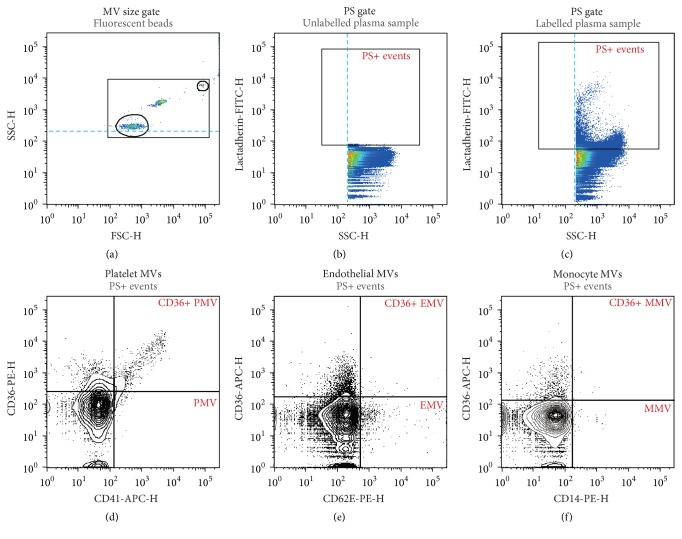
Gating strategy for the flow cytometric microvesicle assay. (a) A 0.1–1.0 *μ*m size gate was defined using silica beads. (b) Unlabelled plasma samples and isotype controls were used to establish gates in the fluorescence channels. (c) MVs were defined as phosphatidylserine expressing (PS+) particles based on their ability to bind lactadherin within the 0.1–1.0 *μ*m gate. (d–f) CD41-APC, CD62E-PE, and CD14-PE antibodies were used to identify MVs of platelet (d), endothelial (e), and monocyte (f) origin, respectively, and their expression of CD36 was investigated using either CD36-PE or CD36-APC antibodies, as appropriate.

Flow cytometric analysis of plasma MV content was performed according to a recently described protocol [19] on a BD FACSAria™ III High Speed Cell Sorter (BD Biosciences, San Jose, CA, USA) at a maximal rate of 2 × 10^4^ events per second until 10^6^ events were collected in total. Data processing was performed as demonstrated in Figure 1. A size gate encompassing approximately 100 nm to 1000 nm was established in FlowJo™ software (v. 10.0.8, Tree Star, Inc., Oregon, USA) using the “Autogate” function around 200 nm and 900 nm fluorescent, size calibrated beads on log-scaled forward scatter height (FSC-H), and side scatter height (SSC-H). In addition, a discriminator was set at 200 on SSC-H to avoid exclusion of the smallest events. MVs were defined as phosphatidylserine positive (PS+) events based on binding of lactadherin-FITC within the established size gate. MVs of platelet origin were defined as CD41 positive events; monocyte MVs (MMVs) were defined as CD14 positive events; endothelial MVs (EMVs) were defined as CD62E positive events, and the expression of CD36 was investigated on PMVs, MMVs, and EMVs.

### 2.6. Data Analysis

All data analyses were conducted in R 3.2.2 (R Core Team, Vienna, Austria). The assumption of normality was tested with Shapiro-Wilk's *W* test for each parameter and visually confirmed. Observations were compared within the respective groups with paired Student's *t*-test or paired Wilcoxon signed ranks test where appropriate. Bivariable correlations between MV subpopulations and anthropometric, biochemical, and clamp parameters were studied using Spearman's ranked correlation coefficients (*r*_*S*_) due to nonnormal distribution of MV parameters. All *p* values reported are two-sided, and statistical significance was defined as *p* < 0.05.

## 3. Results

### 3.1. Characterization of Study Subjects

Demographic characteristics of the subjects were previously described by Magnussen et al. [8] and are summarized in Table 1. Of the thirty-nine subjects enrolled in this study, twenty were assigned to TRT and nineteen to placebo. In the initial assessment, no significant differences existed between TRT and placebo.

### 3.2. Body Composition and Markers for Hepatic Fat Accumulation

Correlations between components of MetSy and MV phenotypes are listed in Table 2. CD36+ MMVs were the only MV subpopulation found to be significantly correlated with BMI (*r*_*S*_ = 0.35, *p* < 0.05), waist circumference (*r*_*S*_ = 0.34, *p* < 0.05), and fat % (*r*_*S*_ = 0.33, *p* < 0.05). No correlations between lean body mass and any MV phenotypes could be reported, however. Positive correlations were identified between MVs and ALT and between GGT and PMVs (*r*_*S*_ = 0.43, *p* < 0.01), CD36+ PMVs (*r*_*S*_ = 0.38, *p* < 0.05), and EMVs (*r*_*S*_ = 0.33, *p* < 0.05). Furthermore, CD36+ MMVs correlated positively with both ALT (*r*_*S*_ = 0.34, *p* < 0.05) and GGT (*r*_*S*_ = 0.42, *p* < 0.01).

### 3.3. Plasma Lipid Profile

Positive correlations were identified between total cholesterol and PMVs (*r*_*S*_ = 0.48, *p* < 0.01) and CD36+ PMVs (*r*_*S*_ = 0.49, *p* < 0.01). Moreover, CD36+ PMVs were positively correlated with LDL cholesterol (*r*_*S*_ = 0.37, *p* < 0.05). Triglycerides correlated positively with MVs (*r*_*S*_ = 0.43, *p* < 0.01), PMVs (*r*_*S*_ = 0.58, *p* < 0.001), CD36+ PMVs (*r*_*S*_ = 0.51, *p* < 0.01), EMVs (*r*_*S*_ = 0.38, *p* < 0.05), CD36+ EMVs (*r*_*S*_ = 0.41, *p* < 0.05), MMVs (*r*_*S*_ = 0.50, *p* < 0.01), and CD36+ MMVs (*r*_*S*_ = 0.45, *p* < 0.01). No significant correlations were identified between HDL and MVs, however.

### 3.4. Insulin Sensitivity

Fasting insulin levels correlated positively with MVs (*r*_*S*_ = 0.36, *p* < 0.05) and MMVs (*r*_*S*_ = 0.41, *p* < 0.05), and both fasting levels of insulin (*r*_*S*_ = 0.54, *p* < 0.001) and C-peptide (*r*_*S*_ = 0.43, *p* < 0.01) correlated positively with CD36+ MMVs. No other significant correlations could be identified between MV phenotypes and parameters associated with insulin sensitivity.

### 3.5. TRT

**Figure 2:**
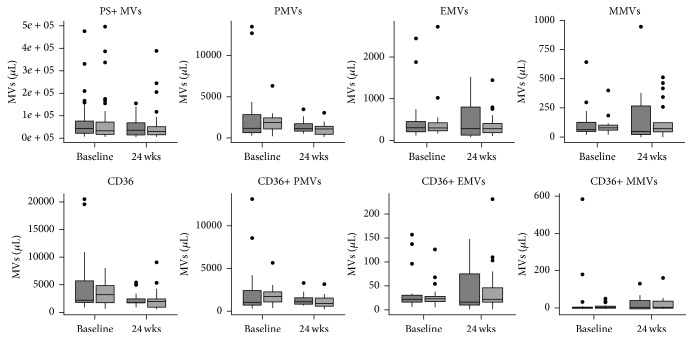
Plasma levels of different MV phenotypes in placebo (*n* = 19, dark grey) and TRT (*n* = 20, light grey) groups at baseline and after 24 weeks of treatment. Outliers are represented as filled circles.

The effect of TRT on the patient population of this study has been described elsewhere [8]. In brief, TRT significantly increased lean body mass (*p* < 0.001) and decreased total fat mass (*p* = 0.0125), fat % (*p* < 0.001), and HDL (*p* = 0.006). No other parameters were affected by TRT. Furthermore, TRT did not affect any of the investigated MV phenotypes (Figure 2).

## 4. Discussion

The results of this study are twofold. First, specific phenotypes of MVs correlated with waist circumference, plasma triglycerides, total cholesterol, and LDL to a certain extent, and putative markers for hepatic fat accumulation, including ALT, and GGT. CD36+ MMVs were of particular significance, and the results of this study are in support of current evidence on the role of monocytes in the pathophysiology of MetSy and T2D. Second, although TRT resulted in improved lean body mass, total fat mass, and fat %, levels of circulating MVs remained unaltered.

By analysing relationships between levels of circulating MVs and components of MetSy and T2D, CD36+ MMVs were revealed to correlate with several measures of body composition. In obesity, adipose tissue monocytes undergo a phenotypic switch and are polarized toward a proinflammatory state [20]. Additionally, distinct differences in monocyte-derived macrophages and inflammatory cytokine profiles have been identified between different adipose tissue depots [5]. Furthermore, expression of CD36 on monocytes is increased in obesity [21], and a large body of evidence suggests that polarization of monocytes is mediated by CD36 [14]. Thus, it is possible that the increased expression of CD36 on the surface of monocytes and their polarization result in increased MV release and that the expression of CD36 on MMVs is equally increased.

Interestingly, PMVs and EMVs were uncorrelated with measures of body composition in the present study. Conversely, several studies have previously identified relationships between PMV and EMV phenotypes and different measures of body composition including BMI, waist circumference, and total fat mass [22]. These associations have previously been attributed to oxidative stress present in obesity [10], as BMI, waist circumference, and total fat mass correlate well with markers for oxidative stress [23]. However, these measures do not take into account the distribution of fat in the different adipose tissue compartments. Furthermore, the different adipose tissue compartments display distinct inflammatory characteristics [5]. Therefore, it would arguably be more accurate to analyse relationships between levels of MVs and the volumes of the different adipose tissue compartments.

In the present study, GGT was found to correlate positively with PMVs, CD36+ PMVs, EMVs, and CD36+ MMVs, and, additionally, ALT correlated positively with CD36+ MMVs. In the presence of hepatic lipid accumulation, it has been reported that hepatic gluconeogenesis and fatty acid oxidation are increased [24], which in turn could be related to liver damage due to a buildup of reactive oxygen species [25]. This results in increased secretion of circulating triglycerides and liver enzymes [26]. GGT and ALT are liver transaminases that are released into circulation in conditions that comprise damage of hepatocytes. One such condition is nonalcoholic fatty liver disease (NAFLD), and ALT and GGT are often used to predict the risk of incident T2D and CVD in these patients [27]. Thus, this result further supports the involvement of monocytes and their expression of CD36 in the pathogenesis of MetSy, T2D, and their related complications.

PMVs and CD36+ PMVs were found to correlate positively with total cholesterol, while only CD36+ PMVs correlated positively with LDL cholesterol. Studies have previously demonstrated that elevated levels of total cholesterol and LDL cholesterol in familial hypercholesterolemia are associated with increased thrombotic activity and platelet function [28]. Recently, plasma levels of total cholesterol have been associated with an increase in mean platelet volume [29], which reflects increased activation, metabolic reactivity, and coagulability [30]. Furthermore, as a consequence of oxidative stress present in MetSy and T2D, LDL becomes oxidized (oxLDL) and accumulates in the circulatory system. oxLDL has previously been implicated in activating platelets through mechanisms involving CD36 [31]. CD36 is expressed abundantly on the surface of platelets, and activation of platelets has been demonstrated to increase surface expression of CD36 by exocytosis of intracellular *α*-granules [32]. Furthermore, oxLDL has previously been demonstrated to increase EMV release [33]; however the finding that EMVs in the present study are unrelated to total cholesterol and LDL cholesterol suggests that this association could be influenced by confounding factors such as diabetes.

Another interesting observation was that all of the investigated MV phenotypes in the present study correlated with circulating triglycerides. One of the main sources of triglycerides in the circulatory system is lipoprotein particles with high concentrations of triglycerides such as very low density lipoproteins (VLDL) [34]. VLDL has been demonstrated to induce platelet hyperreactivity in an in vitro setting [35]. In addition, previous studies have demonstrated that increased levels of EMVs are highly associated with circulating triglycerides [22]. High levels of triglycerides have previously been implicated in impaired endothelial function [36] through mechanisms most likely associated with remnant lipoprotein particles [37] and the production of reactive oxygen species [38]. Taken together, it can be argued that dyslipidaemia affects platelets and endothelial cells and that this is reflected in levels of circulating MVs.

We identified positive associations between MMVs and CD36+ MMVs on the one hand and fasting insulin levels on the other. Additionally, CD36+ MMVs also correlated positively with basal levels of C-peptide. The development of insulin resistance has been highly ascribed to an overabundance of fatty acids as well as ectopic lipid storage [4]. Furthermore, the phenotypic shift observed in monocytes in obesity is thought to contribute to the development of insulin resistance and T2D (reviewed in [39]). This result therefore further supports the involvement of monocytes in the pathogenesis of MetSy and T2D. However, caution should be applied when interpreting results concerning insulin resistance in the participants of this study. Apart from MMVs, no other MV phenotypes were associated with measures of insulin resistance. A possible explanation for this is that the all participants were all prescribed treatment with Metformin, an inhibitor of hepatic glucose output [40]. Through its action in limiting hyperglycaemia, Metformin can therefore be considered a possible confounder that influences the levels of MVs.

In spite of TRT eliciting mild but significant improvements in lean body mass, total fat mass, and fat % in the present study, no significant changes could be observed in any of the studied MV subpopulations. It is important to note that, while TRT elicited mild changes in the abovementioned parameters, most parameters that were found to correlate with the investigated MV phenotypes were unaffected. Thus, it is unlikely that the MV phenotypes included in the present study are affected by TRT.

A number of limitations with this study should be considered. Although the number of participants were determined beforehand to be more than 15 in each group to result in a minimum relevant effect size of 1,3 kg in lean body mass [41] with a 5% probability for type 1 error and 10% probability of a type 2 error, the authors acknowledge that the number of participants in this study is a limitation. Therefore, while some of our findings were statistically significant, several of the null findings might be a consequence of the sample size. Another possible limitation of the present study is that correlations between baseline parameters cannot provide information about causality due to the cross-sectional nature of the analysis and should therefore purely be regarded as hypothesis generating. Finally, due to the highly homogenous nature of the study participants and the lack of a healthy control group, it is uncertain how the results of this study would translate to the overall population, and therefore generalization of these findings should be done with caution.

In conclusion, we demonstrated that the MV phenotypes investigated in this study correlated with several of the components of MetSy and T2D, where CD36+ MMVs were of particular importance. Our findings are therefore in support of the suggested role of monocytes as protagonists in the pathogenesis of the metabolic syndrome and T2D. It can be inferred that the activation of and shift in monocyte polarity from an anti-inflammatory to a proinflammatory phenotype upon increased ectopic fat deposition are mediated by CD36, which in turn leads to increased expression of CD36 on the plasma membrane [5, 14, 20, 21]. This activation accompanied by cell stress caused by the production of reactive oxygen species results in increased budding of the plasma membrane and release of MVs (reviewed in [11]). Taken together, increased numbers of CD36+ MMVs can therefore be proposed as possible blood-based biomarkers for ectopic fat accumulation in obesity, MetSy, and T2D. We additionally demonstrated that TRT did not result in altered levels of the investigated MV phenotypes, even though improvements were seen in lean body mass and total fat mass. It could, however, be argued that the changes in body composition elicited by TRT are mild, and it remains to be demonstrated whether TRT improves ectopic fat deposition and thereby cardiovascular health in patients with T2D. However, it cannot be excluded that the limitations of this study played a role in the latter result. Therefore, the authors of this study suggest that further studies are warranted in which the limitations of the present study are addressed to assess the direct effects of TRT on cardiovascular health in men with T2D.

## Figures and Tables

**Table 1:** Baseline characteristics of study population.

	Placebo (*n* = 19)	Testosterone (*n* = 20)	*p*
Age	59,5 (6,3)	61,4 (6)	0,3367
Smoking status (current/previous/never)^†^	4/9/6	3/12/4	
BMI (kg m^−2^)	30,8 (3,8)	30,6 (4)	0,8879
Waist circumference (cm)	105,8 (10,8)	106,9 (10)	0,7577
Total fat mass (kg)	27,9 (6,5)	26,9 (22,6; 34,8)	0,7837
Lean body mass (kg)	61,7 (7,5)	61,9 (8,9)	0,9531
Systolic blood pressure (mmHg)	138,2 (12,8)	137,7 (16,7)	0,9151
Diastolic blood pressure (mmHg)	81,7 (8,2)	80,5 (11,1)	0,6948
HbA1c (% Hb)	6,50 (6,15; 6,80)	6,52 (0,53)	0,8108
Total cholesterol (mmol L^−1^)	3,8 (1,1)	4,0 (0,7)	0,5511
LDL (mmol L^−1^)	2,2 (0,8)	2,3 (0,7)	0,6904
HDL (mmol L^−1^)	0,9 (0,9; 1)	1,0 (0,2)	0,2057
TG (mmol L^−1^)	1,3 (1,16; 1,58)	1,1 (0,85; 2,05)	0,323
ALT (IU L^−1^)	31,0 (24,5; 43)	35,7 (17,5)	0,844
GGT (IU L^−1^)	39,0 (30; 51,5)	31,0 (23,75; 39,25)	0,1123
Fasting glucose (mmol L^−1^)	6,92 (1,01)	7,14 (1,27)	0,5602
Disposal rate: base	90,0 (12,8)	88,6 (85,39; 109,02)	0,3506
Disposal rate: clamp	179,4 (42,1)	155,9 (136,34; 198,84)	0,5877
ΔRd^‡^	89,4 (40,6)	89,8 (14,9)	0,9655
HGP: base	83,2 (13,2)	89,8 (14,9)	0,1516
HGP: clamp	32,2 (24,57; 38,64)	28,9 (22,02; 40,88)	0,7284
C-peptide: base	1127,8 (318,7)	933,5 (859,25; 1306)	0,422
Insulin: fasting	103,8 (55,9)	89,5 (47,8)	0,4028
Insulin clearance	0,4 (0,1)	0,3 (0,28; 0,33)	0,1912

Data are depicted as mean (SD) or median (Q_25%_; Q_75%_). ^†^Smoking status for 1 person in the testosterone group is unknown. ^‡^Difference in disposal rate at baseline and during clamp.

**Table 2:** Correlations between MV subpopulations and parameters associated with components of MetSy and T2D at baseline.

	BMI	Waist	Total fat mass	Lean body mass	Fat %	HbA1c	Total cholesterol	LDL	HDL	TG	ALT	GGT	Δ*R*_*d*_^†^	HGP: base	HGP: clamp	C-peptide: base	Insulin: fasting	Insulin clearance
Lactadherin+	0,18	0,08	0,09	0,20	0,00	0,30	0,15	0,06	−0,16	0,43^*∗∗*^	0,34^*∗*^	0,27	−0,21	0,12	0,25	0,30	0,36^*∗*^	−0,04
CD41+	0,06	0,09	0,08	0,21	0,00	0,14	0,48^*∗∗*^	0,32	−0,18	0,58^*∗∗∗*^	0,17	0,43^*∗∗*^	−0,07	0,16	0,27	0,14	0,24	−0,19
CD41+/CD36+	−0,01	0,05	0,04	0,18	−0,03	0,03	0,49^*∗∗*^	0,37^*∗*^	−0,11	0,51^*∗∗*^	0,13	0,38^*∗*^	0,00	0,16	0,25	0,08	0,18	−0,16
CD62E+	0,12	0,04	0,08	0,31	-0,06	0,08	0,30	0,14	−0,06	0,38^*∗*^	0,16	0,33^*∗*^	−0,18	0,13	0,14	0,25	0,31	−0,03
CD62E+/CD36+	0,03	−0,04	−0,08	0,27	−0,22	0,18	0,30	0,14	−0,01	0,41^*∗*^	0,01	0,19	0,05	0,19	0,13	0,10	0,17	−0,04
CD14+	0,18	0,10	0,18	0,18	0,14	0,10	0,20	0,09	−0,23	0,50^*∗∗*^	0,19	0,26	−0,19	0,14	0,21	0,30	0,41^*∗*^	−0,19
CD14+/CD36+	0,35^*∗*^	0,34^*∗*^	0,33^*∗*^	0,22	0,28	−0,13	0,22	0,09	−0,21	0,45^*∗∗*^	0,34^*∗*^	0,42^*∗∗*^	−0,26	0,03	0,17	0,43^*∗∗*^	0,54^*∗∗∗*^	−0,11

Spearman's ranked correlation coefficients (*r*_*S*_). ^*∗*^*p* < 0.05; ^*∗∗*^*p* < 0.01; ^*∗∗∗*^*p*< 0.001. ^†^Difference in disposal rate at baseline and during clamp.
